# Morphological and molecular characterization of *Austrodiplostomum compactum* metacercariae in the eyes and brains of fishes from the Ivaí River, Brazil

**DOI:** 10.1590/S1984-29612022021

**Published:** 2022-04-22

**Authors:** Aparecida de Fátima Cracco, Bárbara Scorsim, Alessandra Valéria de Oliveira, Ricardo Massato Takemoto

**Affiliations:** 1 Programa de Pós-graduação em Biologia Comparada, Centro de Ciências Biológicas, Universidade Estadual de Maringá – UEM, Maringá, PR, Brasil; 2 Laboratório de Ictioparasitologia, Núcleo de Pesquisas em Limnologia, Ictiologia e Aquicultura – NUPÉLIA, Centro de Ciências Biológicas, Universidade Estadual de Maringá – UEM, Maringá, PR, Brasil; 3 Programa de pós-graduação em Ecologia de Ambientes Aquáticos Continentais, Centro de Ciências Biológicas, Universidade Estadual de Maringá – UEM, Maringá, PR, Brasil; 4 Departamento de Biotecnologia, Genética e Biologia Celular, Núcleo de Pesquisas em Limnologia, Ictiologia e Aquicultura – NUPÉLIA, Programa de Pós-graduação em Ecologia de Ambientes Aquáticos Continentais, Centro de Ciências Biológicas, Universidade Estadual de Maringá – UEM, Maringá, PR, Brasil

**Keywords:** *COI* gene tree, Diplostomidae, endoparasites, freshwater fish, Loricariidae, Árvore gênica *COI*, Diplostomidae, endoparasitos, peixes de água doce, Loricariidae

## Abstract

*Austrodiplostomum* spp. (Platyhelminthes: Digenea) are endoparasites with a broad geographic distribution in South America. During the larval stage, they parasitize the eyes, brains, muscles, gill, kidneys and swim bladder of a wide variety of fishes. The metacercariae of *Austrodiplostomum* spp. have several morphological characteristics during development, but are very similar among species, which makes it necessary to use molecular tools to contribute to the elucidation during the larval stage. The objective of this study was to perform morphological and molecular analyses of *Austrodiplostomum* sp. found in specimens of *Hypostomus* sourced from the Ivaí River in the state of Paraná, Brazil. Of the 93 analyzed specimens (*H. hermanni* [n = 50], *H. albopunctatus* [n = 9], *Hypostomus* sp. 1 [n = 24], and *Hypostomus* sp. 2 [n = 10]), 60 were parasitized. A total of 577 *Austrodiplostomum* sp. metacercariae was collected from the infected hosts; DNA from seven of these samples was extracted, amplified, and sequenced. The morphological data associated with the genetic distance values and the relationships observed in the *COI* gene tree, indicate that all metacercariae were *A. compactum*. This is the first record of *A. compactum* parasitizing *H. hermanni*, *H. albopunctatus*, *Hypostomus* sp. 1, and *Hypostomus* sp. 2 in the Ivaí River.

## Resumo

*Austrodiplostomum* spp. (Platyhelminthes: Digenea) são endoparasitos com uma ampla distribuição geográfica na América do Sul. Durante a fase larval, parasitam os olhos, cérebros, músculos, brânquias, rins e bexiga natatória de uma grande variedade de peixes. As metacercárias de *Austrodiplostomum* spp. apresentam várias características morfológicas durante o desenvolvimento, as quais são muito semelhantes entre as espécies, o que torna necessário o uso de ferramentas moleculares para contribuir para a elucidação durante a fase larval. O objetivo deste estudo foi realizar análises morfológicas e moleculares de *Austrodiplostomum* sp. encontradas em espécimes de *Hypostomus* provenientes do rio Ivaí, no Paraná, Brasil. Dos 93 espécimes analisados (*H. hermanni* [n = 50], *H*. *albopunctatus* [n = 9], *Hypostomus* sp. 1 [n = 24], e *Hypostomus* sp. 2 [n = 10]), 60 foram parasitados. Um total de 577 metacercárias de *Austrodiplostomum* foram coletadas dos hospedeiros infectados; o DNA de sete dessas amostras foi extraído, amplificado e sequenciado. Os dados morfológicos, associados aos valores de distância genética e as relações observadas na árvore gênica do *COI*, indicam que todas as metacercárias são *A. compactum*. Este é o primeiro registo de *A. compactum* parasitando *H. hermanni, H. albopunctatus*, *Hypostomus* sp. 1, e *Hypostomus* sp. 2 no rio Ivaí.

## Introduction

*Austrodiplostomum* Szidat & Nani, 1951 (Digenea: Diplostomidae) are digenetic trematodes belonging to the phylum Platyhelminthes (Travassos et al., 1928; Takemoto et al., 2009). This genus included only two species: *A. mordax* Szidat & Nani, 1951, found in South America, and *A. compactum* Lutz, 1928 (syn. *A. ostrowskiae* Dronen, 2009) reported in the United States, Mexico, El Salvador, Honduras, Costa Rica, Venezuela, Peru and Brazil (García-Varela et al., 2016; De Núñez, 2017; Sereno-Uribe et al., 2019a).

Digenetic trematodes have a complex life cycle in which they can parasitize three hosts (Kohn et al., 2013). In the adult stage, they are found in the digestive tract of piscivorous birds, such as *Nannopterum brasilianus* (Gmelin, 1789), the definitive hosts of this parasite (Kennedy & Spencer, 2014; García-Varela et al., 2016). During reproduction, the parasite produces eggs that are released into the aquatic environment through the feces of the birds (Travassos et al., 1928). The miracidia emerge from the eggs and penetrate the integument of mollusks of the genus *Biomphalaria* (Preston, 1910): *B. straminea* (Dunker, 1848), *B. glabrata* (Say, 1818), *B. prona* (Martens, 1873), and *B. havanensis* (Pfeiffer, 1839) (Pinto & Melo, 2013; Rosser et al., 2016; De Núñez, 2017).

After morphological transformations, the miracidium gives rise to free-swimming forms of the parasite called cercariae (Travassos et al., 1928). Cercariae can actively infect fish and evolve into metacercariae. The metacercariae of *Austrodiplostomum* spp. are usually found in the eyes, brains, and muscles of fishes (Travassos et al., 1928; Lehun et al., 2020). However, can also be found in the gill, renal parenchyma and swim bladder (Monteiro et al., 2016). The presence of this parasite in the eyes can impair vision, which makes the infected fishes susceptible to predation and facilitates transmission of the parasite to the definitive host, piscivorous birds (Affonso et al., 2017).

In Brazil, *A. compactum* metacercariae are most abundantly found in fishes from the Cichlidae and Sciaenidae families, but they have also been found in Anostomidae, Auchenipteridae, Characidae, Curimatidae, Erythrinidae, Pimelodidae, and Loricariidae (Santos et al., 2012; Ramos et al., 2013). Despite the wide distribution and increasing number of occurrences of *Austrodiplostomum* spp. in fishes (Pelegrini et al., 2019; Acosta et al., 2020; Campos et al., 2020; Ramos et al., 2013, 2020) in Brazilian rivers, there are still sites where studies are scarce, such as the Ivaí River, located entirely in the state of Paraná, Brazil, and is an important tributary of the Paraná River basin.

*Austrodiplostomum* spp. metacercariae have morphological structures at the developmental stage, including an oral sucker, pseudosuckers, pharynx, intestinal caeca, tribocytic organ, and gonads (De Núñez, 2017). However, at this stage, morphological identification is hampered by the similarity of structures between species and by some morphological features that may be absent (De Núñez, 2017). In these cases, the use of molecular techniques is beneficial in the identification process (Sereno-Uribe et al., 2019b; Onaca et al., 2019). The molecular marker cytochrome *c* oxidase subunit 1 (*COI*) is a useful tool for identifying digeneans. Moszczynska et al. (2009) stated that *COI* sequences showed good resolution at the species level, making it a practical target for digeneans; the same is true in a study by Locke et al. (2015), in which *COI* sequences were suitable for the discrimination of *Diplostomum* von Nordmann, 1832 species.

Thus, the aim of this study was to perform morphological and molecular analyses using the *COI* mitochondrial gene to aid the identification of the larval stage of *Austrodiplostomum* sp., collected from four species of the *Hypostomus* Lacépède, 1803 sourced from the Ivaí River.

## Materials and Methods

Specimens of *Hypostomus* (93) were collected at three sites on the left bank of the Ivaí River, located in the municipality of Engenheiro Beltrão-Paraná, Brazil (23°40’15.4″S, 52°09’36.6″W; 23°40’06.3″S, 52°09’31.5″W; and 23°38’57.7″S, 52°09’52.7″W). The collection was authorized by the Instituto Chico Mendes de Biodiversidade (ICMBio), permit number 66135-3, and occurred in May, June, and September 2019 and March 2020. Fishes were captured by gillnets (3.0/5.0/6.0/7.0 cm measured between opposing stretched nodes) that were placed at the stated locations at dusk and collected the following morning, totaling a 12-h exposure. The captured fish were anesthetized with benzocaine and killed according to the Euthanasia Practice Guidelines of the Conselho Nacional de Controle de Experimentação Animal (CONCEA) with permission from the Comissão de Ética no Uso de Animais of the Universidade Estadual de Maringá (CEUA– no. 5073090620). Fishes were identified according to the protocols by Frota et al. (2016) and Zawadzki et al. (2020). During the identification of the hosts, two species were found that were not yet identified at specific level (*Hypostomus* sp.1 and *Hypostomus* sp.2).

After collecting the biometric data of the fishes, the eyes and brains were removed and placed into Petri dishes containing saline solution. Subsequently, they were observed under a stereomicroscope to search for digenetic parasites. For morphological analysis, metacercariae were collected from the eyes of the following fishes: *Hypostomus* sp. 1 (five specimens), *Hypostomus* sp. 2 (five specimens), *H. albopunctatus* (Regan, 1908) (five specimens with an additional three specimens collected from the brains) and *H. hermanni* (Ihering, 1905) (five specimens with an additional four specimens collected from the brains).

The specimens were fixed and stained with carmine according to the method reported by Eiras et al. (2006). Representative specimens of *Austrodiplostomum compactum* were deposited in the Helminthological Collection of the Instituto Oswaldo Cruz (CHIOC), Rio de Janeiro, Brazil (CHIOC: 39707), and the host fishes were deposited in the Ichthyological Collection of the Núcleo de Pesquisas em Limnologia, Ictiologia e Aquicultura (Nupélia), under the numbers: *H. hermanni* (NUP 22655), *H. albopunctatus* (NUP 22659), *Hypostomus* sp.1 (NUP 22656) and *Hypostomus* sp. 2 (NUP 22657).

The measurements recorded for the length and width of the body, as well as structures such as the oral sucker, pharynx, trybocytic organ, and metacercaria gonads, were expressed in millimeters. The images were captured using the optical photographic equipment OPTHD 3.7, attached to a Nikon Eclipse e200 microscope. The prevalence, mean abundance, and mean intensity of parasitic infections in the hosts were calculated according to Bush et al. (1997).

DNA was extracted from metacercariae (n = 7) of *Austrodiplostomum* sp. found in the following host organs: brain (1) and eyes (1) of *H. albopunctatus*, eyes (3) of *H. hermanni*, eyes (1) of *Hypostomus* sp. 1, and eyes (1) of *Hypostomus* sp. 2. The extraction was carried out using the ReliaPrep™ gDNA Tissue Miniprep System kit, following the manufacturer’s instructions. *COI* gene was partially amplified using primers for Plat-diploCOXF:5′-CGTTTRAATTATACGGATCC-3′ and bR:5′AGCATAGTAATMGCAGCAGC-3′ (Moszczynska et al., 2009).

Polymerase chain reaction (PCR) conditions comprised an initial denaturation at 94 °C for 2 min, followed by 35 cycles of 94 °C for 30 s, 50 °C for 30 s, 72 °C for 1 min, and a final elongation cycle at 72 °C for 10 min. The amplicons were verified on a 1% agarose gel by comparing with a 100 bp ladder Invitrogen (lot no. 765323) and purified using a polyethylene glycol protocol (Rosenthal et al., 1993). The samples were sequenced by ACTGene Análises Moleculares Ltda, using the ABI-3500 automated sequencer. Access to genetic heritage was authorized by the Sistema Nacional de Gestão do Patrimônio Genético e do Conhecimento Tradicional Associado (register no. A29B419).

The sequences obtained were edited manually and aligned using the BioEdit 7.2 (Hall, 1999) and MEGA 7.0 (Kumar et al., 2016) software, respectively. The sequence similarity values of the parasites were obtained by comparing the sequences with GenBank data sets using the BLASTn tool. The novel sequences were deposited in GenBank (accession numbers: MT627211; MT632470–MT632475).

To construct the gene tree using the *COI* gene, sequences of *Austrodiplostomum*, *Diplostomum*, and *Tylodelphys* Diesing, 1850 were added from GenBank, and *Australapatemon mclaughlini* Gordy, Locke, Rawlings, Lapierre, Hanington, 2017 was used as an outgroup (Table 1). The gene tree was constructed using the maximum likelihood statistical method with the Randomized Axelerated Maximum Likelihood (RaxML) program (Kozlov et al., 2019). The best nucleotide substitution model (HKY+I+G) was selected based on the Bayesian information criterion using jModelTest 2 (Darriba et al., 2012).

**Table 1:** *COI* sequences available in GenBank used in this study.

Parasite species	Location	Haplotype	GenBank accession number	Reference
*Austrodiplostomum compactum*	-------	H1	MH378899-MH378901	Sereno-Uribe et al. (2019a)
*Austrodiplostomum compactum*	-------	H16	MH378902	Sereno-Uribe et al. (2019a)
*Austrodiplostomum compactum*	-------	H1	MH378903- MH378905	Sereno-Uribe et al. (2019a)
*Austrodiplostomum compactum*	-------	H5	MH378906	Sereno-Uribe et al. (2019a)
*Austrodiplostomum compactum*	-------	H1	MH378907	Sereno-Uribe et al. (2019a)
*Austrodiplostomum compactum*	-------	H17	MH378908	Sereno-Uribe et al. (2019a)
*Austrodiplostomum compactum*	-------	H18	MH378909	Sereno-Uribe et al. (2019a)
*Austrodiplostomum compactum*	-------	H7	MH378910	Sereno-Uribe et al. (2019a)
*Austrodiplostomum compactum*	Honduras	H2	MH378911	Sereno-Uribe et al. (2019a)
*Austrodiplostomum compactum*	-------	H1	MH378912- MH378914	Sereno-Uribe et al. (2019a)
*Austrodiplostomum compactum*	-------	H19	MH378915	Sereno-Uribe et al. (2019a)
*Austrodiplostomum compactum*	-------	H1	MH378916- MH378919	Sereno-Uribe et al. (2019a)
*Austrodiplostomum compactum*	-------	H8	MH378920- MH378921	Sereno-Uribe et al. (2019a)
*Austrodiplostomum compactum*	Mexico	H2	MH378922	Sereno-Uribe et al. (2019a)
*Austrodiplostomum compactum*	-------	H1	MH378923	Sereno-Uribe et al. (2019a)
*Austrodiplostomum compactum*	-------	H7	MH378924	Sereno-Uribe et al. (2019a)
*Austrodiplostomum compactum*	-------	H1	MH378925- MH378927	Sereno-Uribe et al. (2019a)
*Austrodiplostomum compactum*	-------	H2	MH378940	Sereno-Uribe et al. (2019a)
*Austrodiplostomum compactum*	-------	H1	MH378941- MH378949	Sereno-Uribe et al. (2019a)
*Austrodiplostomum compactum*	-------	H8	MH378950	Sereno-Uribe et al. (2019a)
*Austrodiplostomum compactum*	-------	H1	MH378951- MH378953	Sereno-Uribe et al. (2019a)
*Austrodiplostomum compactum*	Brazil	H21	MN179321	Lopez-Hernandez et al. (2019)
*Austrodiplostomum compactum*	Brazil	H20	MN179320	Lopez-Hernandez et al. (2019)
*Austrodiplostomum compactum*	Brazil	H1	MZ323246	Achatz et al. (2022)
*Austrodiplostomum ostrowskiae*	Brazil	H1	KR271025	Locke et al. (2015)
*Austrodiplostomum ostrowskiae*	United States	H4	KR271026	Locke et al. (2015)
*Austrodiplostomum ostrowskiae*	Peru	H15	KR271027	Locke et al. (2015)
*Austrodiplostomum ostrowskiae*	United States	H2	KR271028	Locke et al. (2015)
*Austrodiplostomum ostrowskiae*	-------	H1	KM115890-KM115899	García-Varela et al. (2016)
*Austrodiplostomum ostrowskiae*	-------	H4	KM115884-KM115889	García-Varela et al. (2016)
*Austrodiplostomum ostrowskiae*	United States	H1	KT728783	Rosser et al. (2016)
*Austrodiplostomum ostrowskiae*	United States	H6	KT728787- KT728788	Rosser et al. (2016)
*Austrodiplostomum ostrowskiae*	United States	H6	KT728798	Rosser et al. (2016)
*Austrodiplostomum ostrowskiae*	United States	H1	KT728799	Rosser et al. (2016)
*Austrodiplostomum ostrowskiae*	United States	H6	KT728791	Rosser et al. (2016)
*Austrodiplostomum ostrowskiae*	United States	H1	KT728793	Rosser et al. (2016)
*Austrodiplostomum ostrowskiae*	United States	H4	KT728794	Rosser et al. (2016)
*Austrodiplostomum ostrowskiae*	United States	H9	KT728795	Rosser et al. (2016)
*Austrodiplostomum ostrowskiae*	United States	H2	KT728786	Rosser et al. (2016)
*Austrodiplostomum ostrowskiae*	-------	H1	KM115883	García-Varela et al. (2016)
*Austrodiplostomum ostrowskiae*	Mexico	H2	KM115944	García-Varela et al. (2016)
*Austrodiplostomum ostrowskiae*	El Salvador	H7	KM115967	García-Varela et al. (2016)
*Austrodiplostomum ostrowskiae*	El Salvador	H1	KM115966	García-Varela et al. (2016)
*Austrodiplostomum ostrowskiae*	Mexico	H3	KM115965	García-Varela et al. (2016)
*Austrodiplostomum ostrowskiae*	Mexico	H5	KM115964	García-Varela et al. (2016)
*Austrodiplostomum ostrowskiae*	Mexico	H1	KM115962-KM115963	García-Varela et al. (2016)
*Austrodiplostomum ostrowskiae*	Mexico	H1	KM115900--KM115901	García-Varela et al. (2016)
*Austrodiplostomum ostrowskiae*	Mexico	H1	KM115902- KM115909	García-Varela et al. (2016)
*Austrodiplostomum ostrowskiae*	Venezuela	H1	KM115910- KM115914	García-Varela et al. (2016)
*Austrodiplostomum ostrowskiae*	Mexico	H4	KM115915	García-Varela et al. (2016)
*Austrodiplostomum ostrowskiae*	Mexico	H1	KM115916- KM115917	García-Varela et al. (2016)
*Austrodiplostomum ostrowskiae*	Mexico	H4	KM115918- KM115920	García-Varela et al. (2016)
*Austrodiplostomum ostrowskiae*	Mexico	H1	KM115921-KM115933	García-Varela et al. (2016)
*Austrodiplostomum ostrowskiae*	Mexico	H4	KM115934	García-Varela et al. (2016)
*Austrodiplostomum ostrowskiae*	Mexico	H1	KM115935	García-Varela et al. (2016)
*Austrodiplostomum ostrowskiae*	Mexico	H4	KM115936- KM115937	García-Varela et al. (2016)
*Austrodiplostomum ostrowskiae*	Mexico	H1	KM115938	García-Varela et al. (2016)
*Austrodiplostomum ostrowskiae*	Mexico	H10	KM115939	García-Varela et al. (2016)
*Austrodiplostomum ostrowskiae*	Mexico	H11	KM115940	García-Varela et al. (2016)
*Austrodiplostomum ostrowskiae*	Mexico	H1	KM115941- KM115943	García-Varela et al. (2016)
*Austrodiplostomum ostrowskiae*	Mexico	H12	KM115945	García-Varela et al. (2016)
*Austrodiplostomum ostrowskiae*	Mexico	H5	KM115946	García-Varela et al. (2016)
*Austrodiplostomum ostrowskiae*	Mexico	H1	KM115947	García-Varela et al. (2016)
*Austrodiplostomum ostrowskiae*	Mexico	H8	KM115948	García-Varela et al. (2016)
*Austrodiplostomum ostrowskiae*	Mexico	H1	KM115949- KM115950	García-Varela et al. (2016)
*Austrodiplostomum ostrowskiae*	Mexico	H13	KM115951	García-Varela et al. (2016)
*Austrodiplostomum ostrowskiae*	Mexico	H7	KM115952- KM115953	García-Varela et al. (2016)
*Austrodiplostomum ostrowskiae*	Mexico	H1	KM115954- KM115961	García-Varela et al. (2016)
*Austrodiplostomum ostrowskiae*	Mexico	H14	KM115882	García-Varela et al. (2016)
*Austrodiplostomum ostrowskiae*	United States	H2	MF124271	Blasco-Costa & Locke (2017)
*Austrodiplostomum ostrowskiae*	United States	H2	JX468066	O’Hear et al. (2014)
*Austrodiplostomum mordax*	Argentina	-------	MH378895	Sereno-Uribe et al. (2019a)
*Austrodiplostomum mordax*	Argentina	-------	MH378896	Sereno-Uribe et al. (2019a)
*Austrodiplostomum mordax*	Argentina	-------	MH378897	Sereno-Uribe et al. (2019a)
*Austrodiplostomum mordax*	Argentina	-------	MH378898	Sereno-Uribe et al. (2019a)
*Austrodiplostomum* sp.1	Mexico	-------	MH378928	Sereno-Uribe et al. (2019a)
*Austrodiplostomum* sp.1	United States	-------	KR271029	Locke et al. (2015)
*Austrodiplostomum* sp.1	Mexico	-------	MH378929	Sereno-Uribe et al. (2019a)
*Austrodiplostomum* sp.1	Mexico	-------	MH378930	Sereno-Uribe et al. (2019a)
*Austrodiplostomum* sp.2	United States	-------	KU707940	Rosser et al. (2016)
*Austrodiplostomum* sp.2	United States	-------	KR271030	Locke et al. (2015)
*Austrodiplostomum* sp.2	Mexico	-------	MH378938	Sereno-Uribe et al. (2019a)
*Austrodiplostomum* sp.2	Mexico	-------	MH378939	Sereno-Uribe et al. (2019a)
*Diplostomum* sp. 15	China	-------	KR271126	Locke et al. (2015)
*Diplostomum* sp. 9	Canada	-------	KR271410	Locke et al. (2015)
*Diplostomum* sp. 10	Canada	-------	KR271096	Locke et al. (2015)
*Diplostomum* sp. 17	Canada	-------	KR271131	Locke et al. (2015)
*Diplostomum ardeae*	Canada	-------	KR271033	Locke et al. (2015)
*Diplostomum ardeae*	Porto Rico	-------	MT324592	Locke et al. (2020)
*Diplostomum ardeae*	Porto Rico	-------	MT324592	Locke et al. (2020)
*Diplostomum lunaschiae*	Brazil	-------	MT324620	Locke et al. (2020)
*Diplostomum lunaschiae*	Brazil	-------	MT324621	Locke et al. (2020)
*Diplostomum lunaschiae*	Brazil	-------	MT324623	Locke et al. (2020)
*Diplostomum lunaschiae*	Brazil	-------	MT324626	Locke et al. (2020)
*Tylodelphys* sp. 6	Mexico	-------	MK172796	Sereno-Uribe et al. (2019a)
*Tylodelphys* sp. 6	Mexico	-------	MK172797	Sereno-Uribe et al. (2019a)
*Tylodelphys* sp. 6	Mexico	-------	MK172798	Sereno-Uribe et al. (2019a)
*Tylodelphys* sp. 6	Mexico	-------	MK172799	Sereno-Uribe et al. (2019a)
*Australapatemon mclaughlini*	Canada	-------	KY587406	Gordy et al. (2017)
*Austrodiplostomum* sp. (A1)	Brazil	H3	MT627211	Present study
*Austrodiplostomum* sp. (A2)	Brazil	H1	MT632471	Present study
*Austrodiplostomum* sp. (A3)	Brazil	H1	MT632473	Present study
*Austrodiplostomum* sp. (A4)	Brazil	H1	MT632474	Present study
*Austrodiplostomum* sp. (A5)	Brazil	H1	MT632475	Present study
*Austrodiplostomum* sp. (A6)	Brazil	H1	MT632470	Present study
*Austrodiplostomum* sp. (A7)	Brazil	H2	MT632472	Present study

The Kimura-2-parameter (K2P) distance was calculated between the analyzed species obtained in this study and sequences available in GenBank using MEGA 7.0 software. The groups were formed according to the species identified in the database. The haplotype network was generated using the PopArt program (Leigh & Bryant, 2015), and all sequences available in GenBank for *A. compactum* and *A. ostrowskiae* were used, totaling 155 sequences. Although they were presented as synonyms (De Núñez, 2017), in our analyses, the name *A. ostrowskiae* was kept as it is in the deposit record, precisely to confirm that they are genetically the same species.

## Results

Of the total specimens analyzed (n = 93), 60 were infected and 577 metacercariae were collected from infected eyes and brains. In the eyes, metacercariae were found free in the aqueous and vitreous humor. In the brain, metacercariae were found free, close to the optic nerve and at a lower quantity when compared to the eyes of the same hosts. Only *H. hermanni* and *H. albopunctatus* fishes presented metacercariae in the brain (Table 2).

**Table 2:** *Austrodiplostomum compactum* in *Hypostomus* spp. from the Ivaí River, Engenheiro Beltrão, Paraná, Brazil.

Hosts	N/I	N/E	N/B	P (%)	MA ± SE	MI ± SE
*H. hermanni*	50/38	500	4	76	10. ± 2.43	13.2 ± 2.64
*H. albopunctatus*	09/08	41	7	88.8	4.55 ± 0.98	5.1 ± 0.86
*Hypostomus* sp.1	24/10	19	0	41.6	0.79 ± 0.20	1.9 ± 0.11
*Hypostomus* sp.2	10/04	06	0	40	0.60 ± 0.26	1.5 ± 0.18

(N/I) number of fishes analyzed and number of infected fishes; (N/E) number of metacercariae collected in eyes; (N/B) number of metacercariae collected in brain; (P%) prevalence expressed as a percentage; (MA) mean abundance; (MI) mean intensity; (SE) standard error.

The biometric data of the hosts showed that *H. albopunctatus* had the largest weight and size (Table 3). However, there was no morphological variation between metacercariae of different species, between hosts of the same species or between different collection sites. The amount of metacercariae collected from the eyes and brains of fishes’ species demonstrates that infection can occur at random. Some hosts, regardless of size, may have a greater amount of metacercariae than others, as occurred in the eyes of *H. hermanni*, where 72 metacercariae were collected, while in other species less than 20 metacercariae were found.

**Table 3:** Biometric data of *Hypostomus* spp. (Siluriformes, Loricariidae) collected from the Ivaí River, Engenheiro Beltrão, Paraná, Brazil.

Host	N	SL ± SE (cm)	W ± SE (g)
*H. hermanni*	50	16.15 ± 0.23	128.65 ± 6.16
*H. albopunctatus*	9	23.33 ± 1.75	342.66 ± 61.46
*Hypostomus* sp.1	24	12.95 ± 0.31	67.03 ± 5.47
*Hypostomus* sp.2	10	12.02 ± 0.44	50.53 ± 5.31

Mean values are expressed followed by ± standard error. (N) number of hosts analyzed; (SL) standard length; (W) weight.

**Figure 1:**
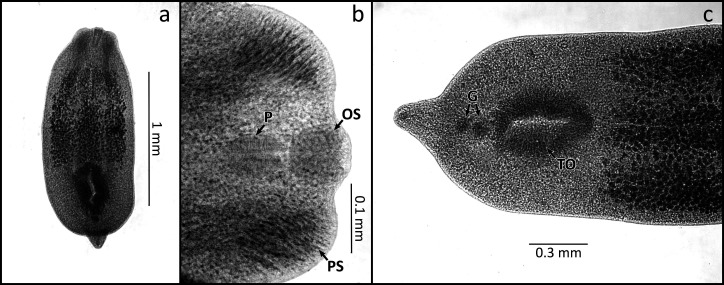
Metacercariae of *Austrodiplostomum compactum* collected in eyes and brains *Hypostomus hermanni* from the Ivaí River - Paraná - Brazil. Metacercariae body (a), forebody; OS = oral sucker, PS = pseudosuckers, P = pharynx (b), hind body; TO = tribocytic organ, G = gonads (c).

The main morphological characteristics and structure measurements of 20 metacercariae specimens collected from the eyes of *Hypostomus* were: Elongated body, slightly concave in the ventral face 1.63 (1.4-1.97) long, 0.59 (0.5-0.75) wide; in relation to the body has a small conical segment in the posterior region; subterminal oral sucker 0.06 (0.04-0.09) long, 0.06 (0.05-0.09) wide; have two pseudosuckers, one on each side of the oral sucker; pharynx 0.05 (0.05-0.07) long, 0.054 (0.04-0.08) wide; esophagus short; intestinal caeca ending near the posterior region; oval tribocytic organ in the posterior half of the body 0.3 (0.1- 0.35) long, 0.17 (0.08-0.22) wide; anterior gonad 0.048 (0.03-0.06) long, 0.046 (0.04-0.06) wide; posterior gonad 0.045 (0.03-0.07) long, 0.045 (0.04-0.06) wide; width glandular cells scattered throughout the body (Figure 1). The metacercariae found in the hosts brains showed no differences in morphological or structural measurements.

**Figure 2:**
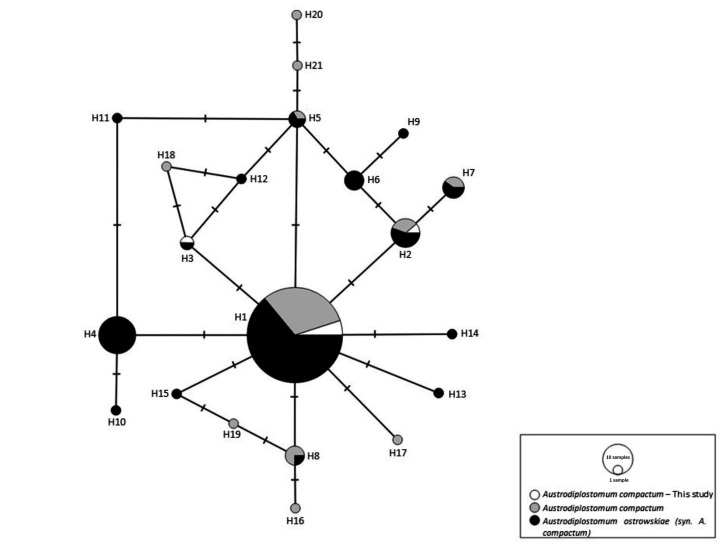
Haplotype network of *Austrodiplostomum compactum* (syn. *A. ostrowskiae*) obtained from GenBank and the sequences obtained in this study.

Sequences of the *COI* region (405 bp) were obtained for seven specimens, totaling three distinct haplotypes in the Ivaí River. Overall, 21 haplotypes were obtained by the analysis of *A. compactum* sequences from GenBank. Considering the sequences obtained in this study, five were allocated to the most common haplotype (H1) (Figure 2). This haplotype was shared by 64.52% of the total analyzed sequences, including samples from other regions of Brazil, Mexico, Venezuela, El Salvador, and the United States. The A1 specimen, which parasitized the eye of *H. albopunctatus*, constituted a different haplotype (H3), and this was shared with one specimen of *A. ostrowskiae* from México. The A7 specimen shared the H2 haplotype with *A. compactum* and *A. ostrowskiae* specimens from México, United States, and Honduras. Haplotypes H1 and H2 were common in both analyzed species, *A. compactum* and *A. ostrowskiae,* thereby, confirming that it is the same species.

Comparisons made between the sequences obtained from the metacercariae with genetic sequences available in GenBank, resulted in values between 99.01% and 100% similarity with *A. compactum* (K2P distance of 0.1% and 0.4%) and *A. ostrowskiae* (K2P distance of 0.2% and 0.4%), respectively (Table 4). In relation to the other *Austrodiplostomum* species available in GenBank, the values of genetic distance ranged from 10.1% to 11.5%. For *Diplostomum* species, the values ranged from 13.6% to 16.6%, whereas those for *Tylodelphys* sp. ranged from 10.6% to 10.9%.

**Table 4:** Values of genetic distance (K2P) between the sequences of the *COI* region obtained from GenBank (8-16), grouped according to the identified species, and sequences of *Austrodiplostomum* sp. obtained in the present study (1-7).

	1	2	3	4	5	6	7	8	9	10	11	12	13	14		15
1. A1																
2. A2	0.0 02															
3. A3	0.002	0.000														
4. A4	0.002	0.000	0.000													
5. A5	0.002	0.000	0.000	0.000												
6. A6	0.002	0.000	0.000	0.000	0.000											
7. A7	0.005	0.002	0.002	0.002	0.002	0.002										
8. *Austrodiplostomum* sp. 1	0.104	0.101	0.101	0.101	0.101	0.101	0.104									
9. *Austrodiplostomum* sp. 2	0.108	0.105	0.105	0.105	0.105	0.105	0.108	0.108								
10. Austrodiplostomum compactum*	0.004	0.001	0.001	0.001	0.001	0.001	0.002	0.103	0.106							
11. *Austrodiplostomum ostrowskiae*	0.004	0.002	0.002	0.002	0.002	0.002	0.002	0.102	0.105	0.002						
12. *Autrodiplostomum mordax*	0.109	0.112	0.112	0.112	0.112	0.112	0.115	0.134	0.132	0.113	0.114					
13. *Diplostomum* sp.	0.156	0.156	0.156	0.156	0.156	0.156	0.153	0.165	0.184	0.154	0.154	0.167				
14. *Diplostomum ardeae*	0.136	0.139	0.139	0.139	0.139	0.139	0.136	0.160	0.181	0.138	0.137	0.161	0.129			
15. *Diplostomum lunaschiae*	0.163	0.166	0.166	0.166	0.166	0.166	0.163	0.180	0.187	0.165	0.164	0.174	0.141	0.081		
16. *Tylodelphys* sp.	0.106	0.109	0.109	0.109	0.109	0.109	0.106	0.139	0.138	0.108	0.108	0.144	0.153	0.137	0.145	

* *A. compactum* (syn. *A. ostrowskiae*).

**Figure 3:**
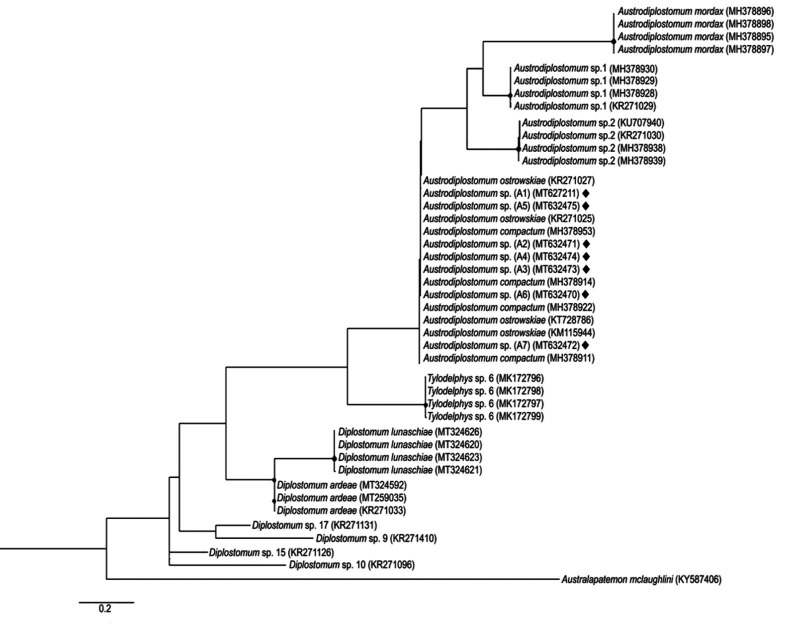
Maximum-likelihood gene tree constructed using the *COI* gene sequences. *Australapatemon mclaughlini* was used as an outgroup. ♦ = Sequences obtained from *Austrodiplostomum* sp. collected in fishes from the Ivaí River.

The gene tree presented in Figure 3 shows that the sequences derived from the samples extracted from the Ivaí River (highlighted by lozenge) as well as, sequences from *A. compactum* (syn. *A. ostrowskiae*), were allocated to a single clade. Interestingly, the clade was close to another cluster formed by a different species of *Austrodiplostomum,* whereas the species of *Tylodelphys* and *Diplostomum* formed distinct clusters.

## Discussion

The morphological data associated with the partial sequences of the *COI* gene (the distance values and the relationships observed in the gene tree) indicate that all metacercariae in this study were *A. compactum*. Moreover, the data corroborate with the morphological data described by De Núñez (2017) that assumes synonymy between *A. compactum* and *A. ostrowskia*e. *COI* gene provided important information for the identification of digenean at the species level, and this led to the generation of seven new sequences. Among the *Hypostomus* species analyzed, three parasite haplotypes were found, the most frequent (H1) being recorded in all species and occurring in both eyes and brains.

The genetic distance values generated between the analyzed sequences were similar to those found in *A. compactum*. In contrast, the values found in relation to the other species analyzed were higher than 10%, and similar to the values found among individuals belonging to different genera. For example, the distance value obtained between A3 and *A. mordax* was 11%, whereas between *Diplostomum lunaschiae* Locke, Drago, Núñez, Souza, Takemoto, 2020 and *Tylodelphys* was 14%. According to Hebert et al. (2003), when comparing *COI* gene sequences in the case of animals, values greater than 2% of the distance are indicative of different species.

The metacercariae of *A. compactum* are considered generalists and have a wide geographical distribution (Ramos et al., 2013). In Brazil, metacercariae have been reported in hydrographic basins belonging to the states of Tocantins, Amazonas, Minas Gerais, São Paulo, Santa Catarina, and Paraná, and are found in the eyes of many different fishes that have been recorded in lists of parasite-host interactions (Ramos et al., 2013; Lehun et al., 2020). However, few studies have addressed the presence of *A. compactum* in *Hypostomus*. This digenean has been detected in the eyes of the following fishes: *H. regani* in the upper Paraná River floodplain (Yamada et al., 2008); *H. affinis* (Steindachner, 1877) in the Guandu River, state of Rio de Janeiro (Azevedo et al., 2010); and *H. ancistroides* (Ihering, 1911), *H*. *iheringii* (Regan, 1908), *H. margaritifer* (Regan, 1908), *H*. *strigaticeps* (Regan, 1908), and *Hypostomus* sp. in the Paranapanema River, state of São Paulo (Zica et al., 2011).

In addition to the wide distribution, *A. compactum* can also co-occur sharing the eye lens of fish, with *A. mordax* and Diplostomidae gen. sp. as reported in the studies by Pelegrini et al. (2021) carried out with *H. regani* collected in the Tietê-Batalha drainage basin in the state of São Paulo. Despite this, we found only *A. compactum* in the *Hypostomus* of the Ivaí River.

In the present study, the prevalence of *A. compactum* in *H. hermanni, H. albopunctatus*, *Hypostomus* sp. 1, and *Hypostomus* sp. 2 fishes sourced from the Ivaí River ranged from 40% to 88.8%, which is higher than the prevalence of the parasite in *Hoplias malabaricus* (Bloch, 1794), which was shown to be 11.11%, furthermore the prevalence of *A. compactum* found in *Plagioscion squamosissimus* (Heckel, 1840) sourced from the upper Paraná River floodplain, was found to be 95.06% (Machado et al., 2005). In the present study, the mean intensity showed that 13.26 parasites and 72 metacercariae were found in the eyes of a single individual of the *H. hermanni* species. Therefore, the infection rate by *A. compactum* metacercariae is frequently high as stated by Zica et al. (2009); however, our results show that the infection rate of this parasite in Loricariidae fishes may vary in different geographic locations.

In conclusion, this study contributes to the knowledge of the diversity of the parasitic fauna present in the Ivaí River, especially regarding the geographical distribution of *Austrodiplostomum*. In addition, new hosts have been found, such as *Hypostomus* sp. 1 and *Hypostomus* sp. 2. Further studies are required to monitor the occurrence of this digenean in fishes.
